# GLUT4 localisation with the plasma membrane is unaffected by an increase in plasma free fatty acid availability

**DOI:** 10.1186/s12944-024-02079-z

**Published:** 2024-04-02

**Authors:** J. S. Barrett, J. A. Strauss, L. S. Chow, S. O. Shepherd, A. J. M. Wagenmakers, Y. Wang

**Affiliations:** 1 Research Institute for Sport & Exercise Sciences, Liverpool John Moores University, Tom Reilly Building, Byrom Street, Liverpool, L3 3AF UK; 2 Department of Medicine, University of Minnesota, Minneapolis, MN USA; 3 Discovery Sciences, AstraZeneca R&D, Cambridge Science Park, Milton Road, Cambridge, CB4 0WG UK

**Keywords:** Lipid infusion, GLUT4 translocation, Skeletal muscle

## Abstract

**Background:**

Insulin-stimulated glucose uptake into skeletal muscle occurs via translocation of GLUT4 from intracellular storage vesicles to the plasma membrane. Elevated free fatty acid (FFA) availability via a lipid infusion reduces glucose disposal, but this occurs in the absence of impaired proximal insulin signalling. Whether GLUT4 localisation to the plasma membrane is subsequently affected by elevated FFA availability is not known.

**Methods:**

Trained (*n* = 11) and sedentary (*n* = 10) individuals, matched for age, sex and body mass index, received either a 6 h lipid or glycerol infusion in the setting of a concurrent hyperinsulinaemic-euglycaemic clamp. Sequential muscle biopsies (0, 2 and 6 h) were analysed for GLUT4 membrane localisation and microvesicle size and distribution using immunofluorescence microscopy.

**Results:**

At baseline, trained individuals had more small GLUT4 spots at the plasma membrane, whereas sedentary individuals had larger GLUT4 spots. GLUT4 localisation with the plasma membrane increased at 2 h (*P* = 0.04) of the hyperinsulinemic-euglycemic clamp, and remained elevated until 6 h, with no differences between groups or infusion type. The number of GLUT4 spots was unchanged at 2 h of infusion. However, from 2 to 6 h there was a decrease in the number of small GLUT4 spots at the plasma membrane (*P* = 0.047), with no differences between groups or infusion type.

**Conclusion:**

GLUT4 localisation with the plasma membrane increases during a hyperinsulinemic-euglycemic clamp, but this is not altered by elevated FFA availability. GLUT4 appears to disperse from small GLUT4 clusters located at the plasma membrane to support glucose uptake during a hyperinsulinaemic-euglycaemic clamp.

## Introduction

In healthy individuals, the physiological increase in insulin following a meal is a potent stimulus for plasma glucose uptake into skeletal muscle [1]. Four hours following oral ingestion of 92 g of glucose, leg glucose uptake is reported to increase three-fold [2], and during a hyperinsulinaemic-euglycaemic clamp leg glucose uptake is elevated approximately five-fold [3]. Approximately ~ 80% of glucose removed from the circulation during a hyperinsulinaemic-euglycaemic clamp in insulin sensitive individuals enters skeletal muscle [4, 5]. This makes skeletal muscle a key determinant of glucose homeostasis, with dysregulation of glucose uptake into muscle having implications for the development of insulin resistance.

Glucose uptake into skeletal muscle occurs predominantly through facilitated diffusion, where glucose transporter proteins play a fundamental role. In total, there are 14 identified glucose transporter isoforms [6], of which, glucose transporter 4 (GLUT4) is the predominant insulin-responsive isoform required for glucose uptake into skeletal muscle [7]. At rest, GLUT4 resides in intracellular cytosolic micro-vesicles (GLUT4 storage vesicles; GSV), but in response to increases in plasma insulin concentrations or muscle contraction an increased number of subsarcolemmal GSV’s merge with the plasma membrane. Early studies used density gradient centrifugation methods to isolate pure plasma membrane fractions and subsequently measure the protein expression of GLUT4. Using this approach, Guma et al. [8] reported a 60% increase in plasma membrane GLUT4 content 30–40 min after the start of a hyperinsulinaemic-euglycaemic clamp.

However, fractionation methods (aside from the potential cross-contamination issues) preclude the ability to understand the cellular distribution of GLUT4. To overcome these issues, electron microscopy or fluorescence microscopy of immuno-stained GLUT4 in muscle fibres have provided both quantitative and spatial information regarding the location of GLUT4 vesicles during basal, insulin-stimulated, and contraction-stimulated states [9]. Studies using both confocal immunofluorescence microscopy and electron microscopy combined with immuno-gold labelling in whole single fibres of rat soleus muscle have shown that GLUT4 is present in the trans-Golgi network (TGN) membranes, endosomal membranes and GSV’s [9–12]. Studies using immunofluorescence microscopy in muscle fibres of rodents have defined GSV’s in the TGN as having a diameter > 1 µm [11]. Interestingly, electron microscopy images have shown that endosomes appear smaller than TGN stores [9], but larger than GSV’s that are reportedly as small as 40 nm [12]. In mice, in vivo methods have been developed where mice express GLUT4-HA that can confirm GLUT4 insertion into the plasma membrane [13, 14], however the chronic expression of tagged-GLUT4 in human models is not possible. Bradley et al*.* [15]*,* developed an immunofluorescence microscopy method to visualize changes in the subcellular distribution and content of GLUT4 in response to oral glucose ingestion and exercise. This study demonstrated a measurable increase in GLUT4 co-localisation with the plasma membrane in response to both glucose ingestion and exercise, alongside a reduction of GLUT4 from large and small clusters [15]. Thus, using this immunofluorescence microscopy method it appears that both exercise and glucose ingestion (separately) stimulate net GLUT4 translocation to the plasma membrane. Missing from this work, however, was whether fibre type differences in GLUT4 distribution and translocation exist.

Elevated plasma free fatty acid (FFA) and triglyceride concentrations give rise to lipid accumulation in skeletal muscle (intramuscular triglyceride; IMTG) in obesity and type 2 diabetes, which is subsequently linked to the development of insulin resistance [16]. More specifically, it is the accumulation of lipid metabolites, such as diacylglycerols and ceramides, which directly impact insulin signalling leading to impaired insulin-stimulated glucose uptake in obese individuals and type 2 diabetes patients [17, 18]. Lipid infusion is a well-established model of lipid-induced insulin resistance, and specifically causes a reduction in peripheral glucose uptake when lipid is infused alongside a hyperinsulinaemic-euglycaemic clamp [19, 20]. Importantly, 2 h of lipid infusion does reduce glucose disposal, but continuing the lipid infusion for 4 to 6 h does eventually lead to a decrease in glucose disposal rates which is similar in trained and sedentary individuals [19, 21, 22]. In response to a 6 h lipid infusion, IMTG content increases independent of training status [22]. However, in the same samples Chow et al*.* [22]*,* reported that sedentary individuals also accumulated diacylglycerol. Interestingly, despite reduced glucose disposal, no changes in the phosphorylation or activation of key insulin signalling components, including insulin receptor substrate (IRS)-1 tyrosine, IRS-1–associated phosphatidylinositol (PI) 3-kinase, Akt, and AS160 was observed [21]. This leads us to question whether the suppression of glucose disposal in response to a lipid infusion impacts the availability of GLUT4 at the plasma membrane.

The aim of the present study was to visualize the co-localisation of GLUT4 to the plasma membrane in human skeletal muscle in response to an Intralipid™ infusion that has previously been shown to reduce glucose uptake [21, 23]. Specifically, we investigated the changes in GLUT4 colocalization to the plasma membrane marker dystrophin and the changes in location and number of both large and small GLUT4 spots that occurs following 2 h and 6 h of either a glycerol or an Intralipid™ infusion alongside a concomitant hyperinsulinaemic-euglycaemic clamp. We tested the hypothesis that GLUT4 colocalization to the plasma membrane would increase at 2 h of either a glycerol or Intralipid™ infusion but would be reduced after 6 h of the Intralipid™ infusion only.

## Methods

### Participants and ethical approval

The muscle samples used in the present study were collected as part of a previous study and therefore the process of recruitment and study protocol have already been described in detail [21, 22]. The current study included 11 healthy lean trained individuals and 10 sedentary individuals that were recruited and matched for sex, age (± 5 years) and BMI (± 1.5 kg/m^2^). Subject characteristics for each group are presented in Table 1. The viability of the samples from two participants (both trained individuals; one from the lipid infusion group and one from the glycerol group) were compromised (due to frost damage) and were not included in the final analysis (9 trained, 10 untrained). Sedentary individuals participated in 30 min or less of active exercise per week, whereas trained individuals participated in a regular running program (≥ 45 min/day, ≥ 5 days/wk). Training was self-reported using the short form International Physical Activity Questionnaire, a validated physical activity questionnaire [24], and fitness level was documented by VO2max testing [21]. The study protocol was approved by the University of Minnesota Institutional Review Board and informed consent was obtained from all participants.

**Table 1:** Baseline characteristics of trained and sedentary participants

	**Trained (*n* = 11)**	**Sedentary (*n* = 10)**	***P* value**
Sex (males/females)	6/5	4/6	0.98
Age (years)	23 ± 1	21 ± 1	0.26
BMI (kg. m^−2^)	22.2 ± 0.6	21.3 ± 0.6	0.31
FFM (kg)	50.8 ± 3.7	40.9 ± 2.3	0.04
Body fat (%)	19.9 ± 2.0	27.4 ± 3.5	0.07
VO_2 max_ (ml. kg^−1^.min^−1^)	47.8 ± 2.0	38.0 ± 1.6	< 0.01
Baseline GIR (μmol glucose infused.kg.FFM^−1^.min^−1^)	66.1 ± 4.7	48.3 ± 5.7	0.03
FFA at end of 6 h lipid infusion (μmol. l^−1^)	600 ± 86	932 ± 105	0.03

Data are the mean ± SEM

*FFM* Free fat mass, *GIR* Glucose infusion rate

### Study protocol

The study protocol has been described in detail previously [21, 22]. Briefly, pre-screening assessments were carried out of body composition (dual-energy X-ray absorptiometry), maximal aerobic fitness (VO_2max_) and insulin sensitivity (3 h hyperinsulinaemic euglycemic clamp). For the hyperinsulinaemic enuglycaemic clamp, insulin was infused [1.5 mU.kg FFM^−1^.min^−1^] alongside a potassium infusion (KPO4 at 50 ml.h^−1^). At the same time a glucose infusion was started (dextrose 20%), and blood glucose was measured every 10 min using a bedside monitor (Analox model GM9D; Analox Instruments, Lunenburg, MA) with the glucose infusion rate subsequently titrated to maintain blood glucose in the range 4.7–5.3 mmol.L^−1^. On a separate day, participants attended the Masonic Clinical Research Unit (MCRU) at the University of Minnesota and consumed a standard evening meal (41% carbohydrate, 32% fat and 27% protein) before remaining on bed rest at the unit overnight until study completion the following day. After an overnight fast, participants underwent either a 6 h lipid infusion (20% Intralipid® at 90 ml.h^−1^ [Baxter, Deerfield, IL, USA]) or 6 h glycerol infusion (2.25 g. 100 ml^−1^ at 90 ml.h^−1^) concurrent with a hyperinsulinemic euglycemic clamp (insulin, 1.5 mU.kg FFM^−1^.min^−1^; KPO4 at 50 ml.h^−1^; dextrose 20% titrated to keep glucose at 4.7–5.3 mmol.L^−1^). Intralipid® was mainly comprised of the following fatty acids: linoleic acid (44–62%), oleic acid (19–30%), palmitic acid (7–14%), linolenic acid (4–11%) and stearic acid (1.4–5.55). The glycerol infusion matched the glycerol content of the lipid infusion to limit the effect of the lipid infusion on FFA elevation [22]. Muscle biopsies were obtained prior to the initiation of the infusion (Bx1), at 120 min (Bx2) and at 360 min (Bx3) of infusion. Each muscle biopsy was dissected free of fat and connective tissue before being embedded in Tissue-Tek OCT Compound (Sakura Finetek Europe, Alphen aan de Rijn, The Netherlands) and frozen in liquid nitrogen-cooled isopentane for immunohistochemical analyses.

### Muscle analysis

The immunohistochemistry staining protocol has been previously reported by Bradley et al*.* (2014). Briefly, serial 5 µm cryosections were cut at -30 °C and transferred to ethanol-cleaned glass slides and fixed and permeabilized in 75% acetone with 25% ethanol for 5 min [25]. Slides were then washed 3 times for 5 min in phosphate-buffered saline (PBS, 137 mmol/L sodium chloride, 3 mmol/L potassium chloride, 8 mmol/L sodium phosphate dibasic, 3 mmol/L potassium phosphate monobasic). The primary antibody targeting GLUT4 (rabbit IgG, ab216661, Abcam, Cambridge, UK) was applied to the sections at a dilution of 1:200 in 5% normal goat serum (ThermoFisher) and was incubated at room temperature for 2 h. The GLUT4 antibody was combined with an antibody targeting dystrophin (D8168, Sigma Aldrich, Dorset UK), to visualise the plasma membrane, and an antibody targeting myosin heavy chain for slow twitch fibres to visualise type I fibres (A4.840, MHC1; mouse IgM,). Following primary antibody incubation, slides were then washed 3 times for 5 min in PBS. Secondary antibodies were applied to the slides for 30 min at room temperature. The GLUT4 antibody was targeted with goat anti-rabbit IgG 488 (A11008), dystrophin with goat anti-mouse IgG_2b_ 546 (A21145) and MHC1 with goat anti-mouse IgM 633 (A21046, Invitrogen, Paisley, UK). Following secondary antibody incubation, slides were washed 3 times for 5 min in PBS and coverslips were mounted with 20 µL mowiol mounting medium [6 g glycerol, 2.4 g mowiol 4–88, and 0.026 g 1,4-Diazabicyclo [2.2.2] octane (DABCO) dissolved in 18 mL 0.2 M Tris buffer (pH 8.5) (All reagents were purchased from Sigma Aldrich, St Louis, MO)] and sealed with nail varnish. Before any colocalization analysis was undertaken, several control experiments were performed (as described previously [25]). These included confirmation of the absence of bleed-through of fluorophores in opposing channels when single staining with GLUT4 or dystrophin was performed, and checking for non-specific secondary antibody binding, and sample autofluorescence.

### Image capture, processing, and analysis

Cross-sectional orientated images were captured using an inverted confocal microscope (Zeiss LSM710; Carl Zeiss AG, Oberkochen, Germany) with a 63 × 1.4 NA oil immersion objective at 1.1 digital zoom. The Alexa Fluor 488 fluorophore was excited with an argon laser, whereas the Alexa Fluor 546 and 633 fluorophores were excited with a helium–neon laser. The objective and magnification used ensured that a single fibre was captured per image, and each imaged fibre was chosen at random only considering the fibre type and not the GLUT4 stain.

Image analysis of GLUT4 content was undertaken using Image-Pro Plus 5.1 software (Media Cybernetics, Bethesda, MD, USA). For each participant, at least 30 images per time-point were taken. Five participants out of the 19 individuals only had samples for two out of the three time points. Therefore, in total there was 204 type I fibres and 201 type II fibres analysed for the lipid infusion group, and 279 type I fibres and 307 type II fibres analysed for the glycerol lipid infusion group.

Fibre type specific GLUT4 content was determined by measuring the fluorescence intensity of the GLUT4 stain. When assessing fibre specific GLUT4 content, fibres stained positively for myosin heavy chain type I were classified as type I fibres, whereas those with no staining were classified as type II fibres. For image analysis of GLUT4 co-localisation to the plasma membrane, Pearson’s correlation coefficient was carried out between the GLUT4 stain and dystrophin border. For quantitation of GLUT4 in the plasma membrane layer (dystrophin-stained region) and in the five 1 μm intracellular layers below the plasma membrane, image segmentation and measurements was carried out in MATLAB (R2020a, The MathWorks Inc., Natick, MA) using a same method as previously used by Bradley et al*.* (2014). Briefly, the analysis algorithm separated the fibres in the dystrophin image using the active contour, or snake, approach [26] to approximately find the mid-point of the plasma membrane. A distance map from the contour then generated a 3-pixel thick region to cover the dystrophin-stained region and was designated the plasma membrane layer. Subsequently, five 1 μm thick layers were generated inside the fibre, again using the distance map. To identify spots from background staining, we used Otsu’s thresholding for each participant. To then distinguish between large and small spots, threshold limits were set for the spot sizes detected (large spots: > 1 μm or small spots: < 1 μm diameter, as in [9–12, 15, 25].

### Statistical analysis

Statistical analysis was carried out in SPSS All analyses were performed using statistical analysis software (SPSS for Mac version 26.0; SPSS, Chicago, IL, USA). Multiple group comparisons to assess GLUT4 protein expression, co-localization and clusters were performed: between: (i) lipid and glycerol infusion groups, (ii) trained and sedentary individuals, (iii) type 1 and type 2 fibres, (iv) time points, and (v) layers for cluster analysis. Linear mixed effects models, with random intercepts to account for repeated measurements within subjects, were used to examine group differences, as well as differences over time of the infusion and between fibre types. Pairwise differences between biopsies were performed using post hoc tests. *P* < 0.05 was considered statistically significant.

## Results

### Effect of lipid or glycerol infusion on glucose infusion rate

**Fig. 1:**
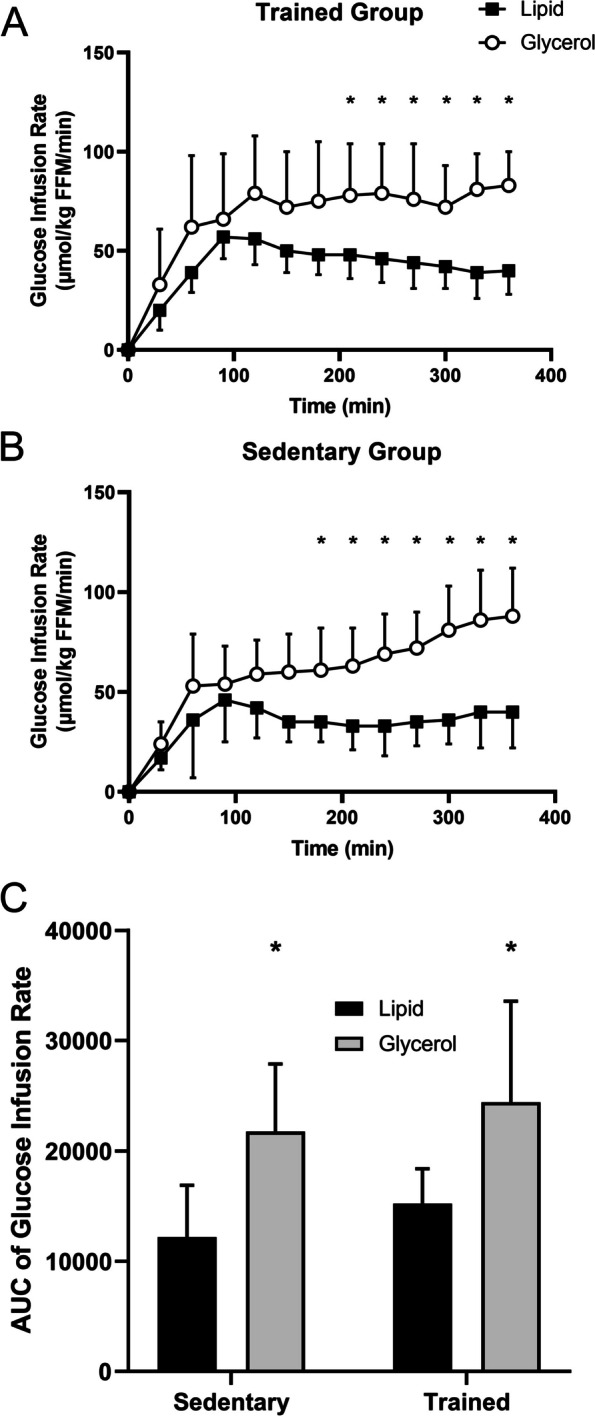
Glucose infusion rate during a 6-h hyperinsulinemic-euglycemic clamp with concurrent lipid or glycerol infusion. The glucose infusion rate needed to maintain euglycemia during the 6-h clamp was higher in the glycerol group compared to the lipid group in both the sedentary (**A**) and trained (**B**) individuals

In response to the lipid infusion, both the sedentary and trained groups exhibited a decreased glucose infusion rate compared to the glycerol control (*P* < 0.05); -54% and -52%, respectively (Fig. 1a and b). In the sedentary group, the divergence in glucose infusion rate between the lipid and glycerol infusions became significant at 180 min (Fig. 1b), whereas the divergence between the two infusions in the trained group became significant at 210 min (Fig. 1a). The difference in the glucose infusion rate AUC between the glycerol and lipid infusion conditions was similar for the sedentary and trained groups (-44% and -38%, respectively; *P* < 0.05; Fig. 1c).

### Protein content and location of GLUT4 in human skeletal muscle at baseline

**Fig. 2:**
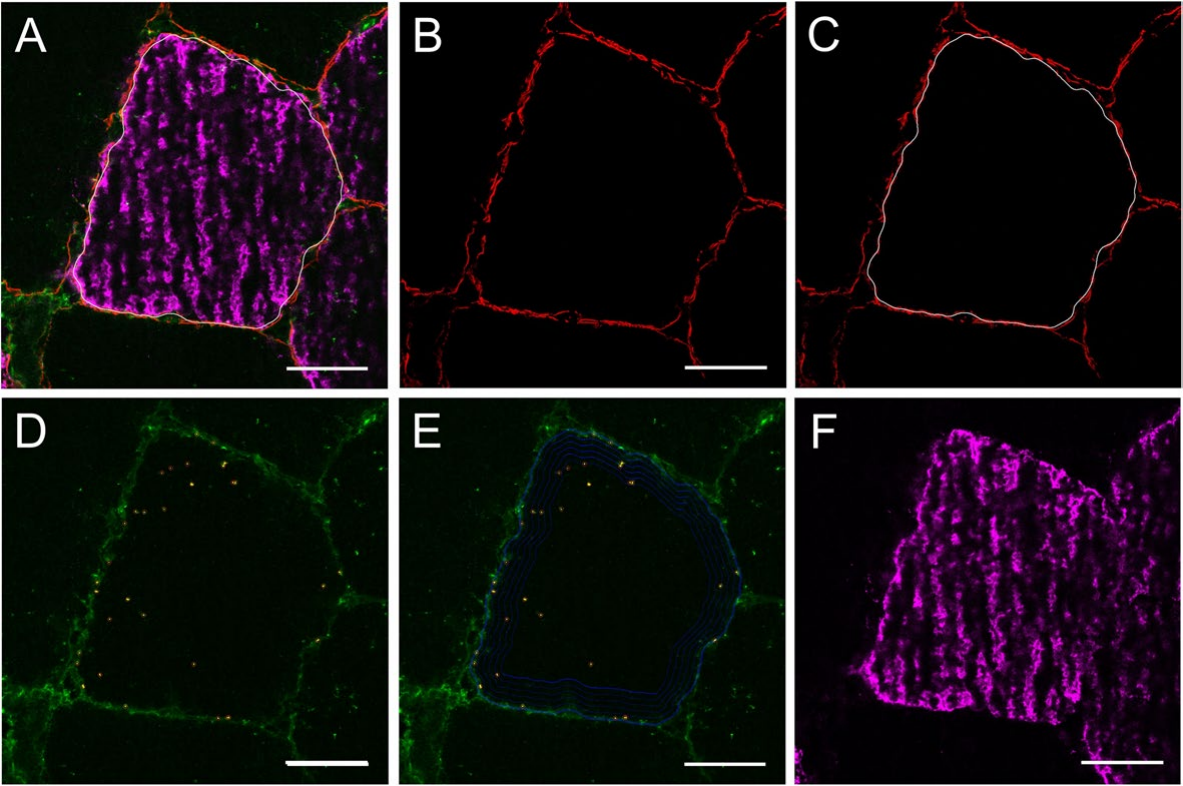
Representative immunofluorescence microscopy image detailing the dystrophin border identification identified using the red dystrophin stain (**A**). Image **B** shows the dystrophin stain alone, which was used to identify the cell boarder carried out in MATLAB (**C**). Image **D** shows the GLUT4 stain (green) including the spots identified by MATLAB analysis in yellow, and this is replicated in E but with the inclusion of the 5 × 1 μm concentric rings that follow the contours of the PM as defined by dystrophin stain. The magenta staining within image A and F represents fibre type staining of MHC1. Scale bar represents 25 μm

Concurrent with previous findings [15, 25], GLUT4 staining in human skeletal muscle revealed both large clusters and small spots throughout the cell (Fig. 2d and e). Both large GLUT4 clusters and small GLUT4 spots can be seen close to, and incorporated within the plasma membrane (stained in red with dystrophin in Fig. 2b and c). However, in contrast to Bradley et al*.,* (2014; 2015), we observed noticeably less spots within all images.

**Fig. 3:**
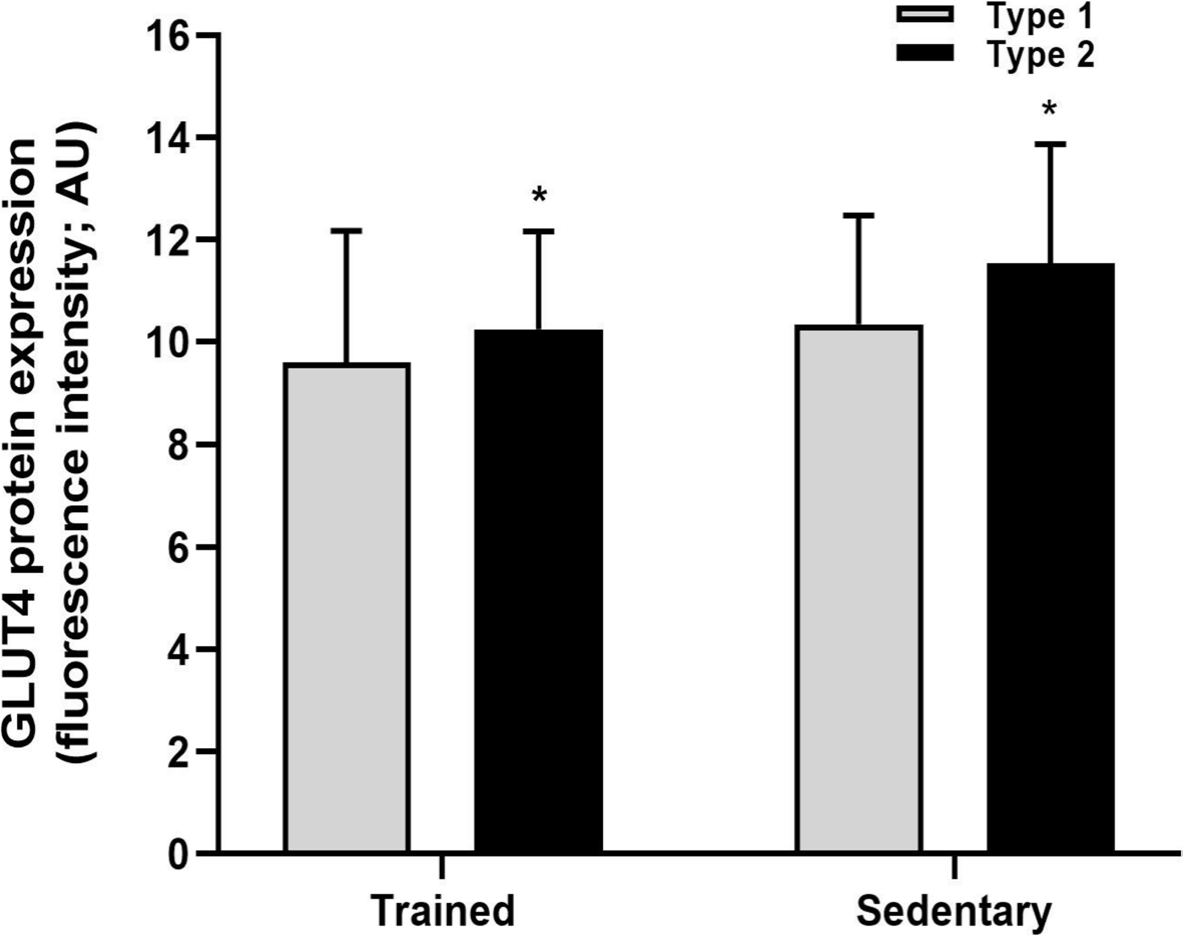
Total GLUT4 fluorescence intensity in type 1 (grey) and type 2 fibres (black) in the basal state. Data are mean ± SD. ^*^Main fibre type effect; *P* = 0.001

**Fig. 4:**
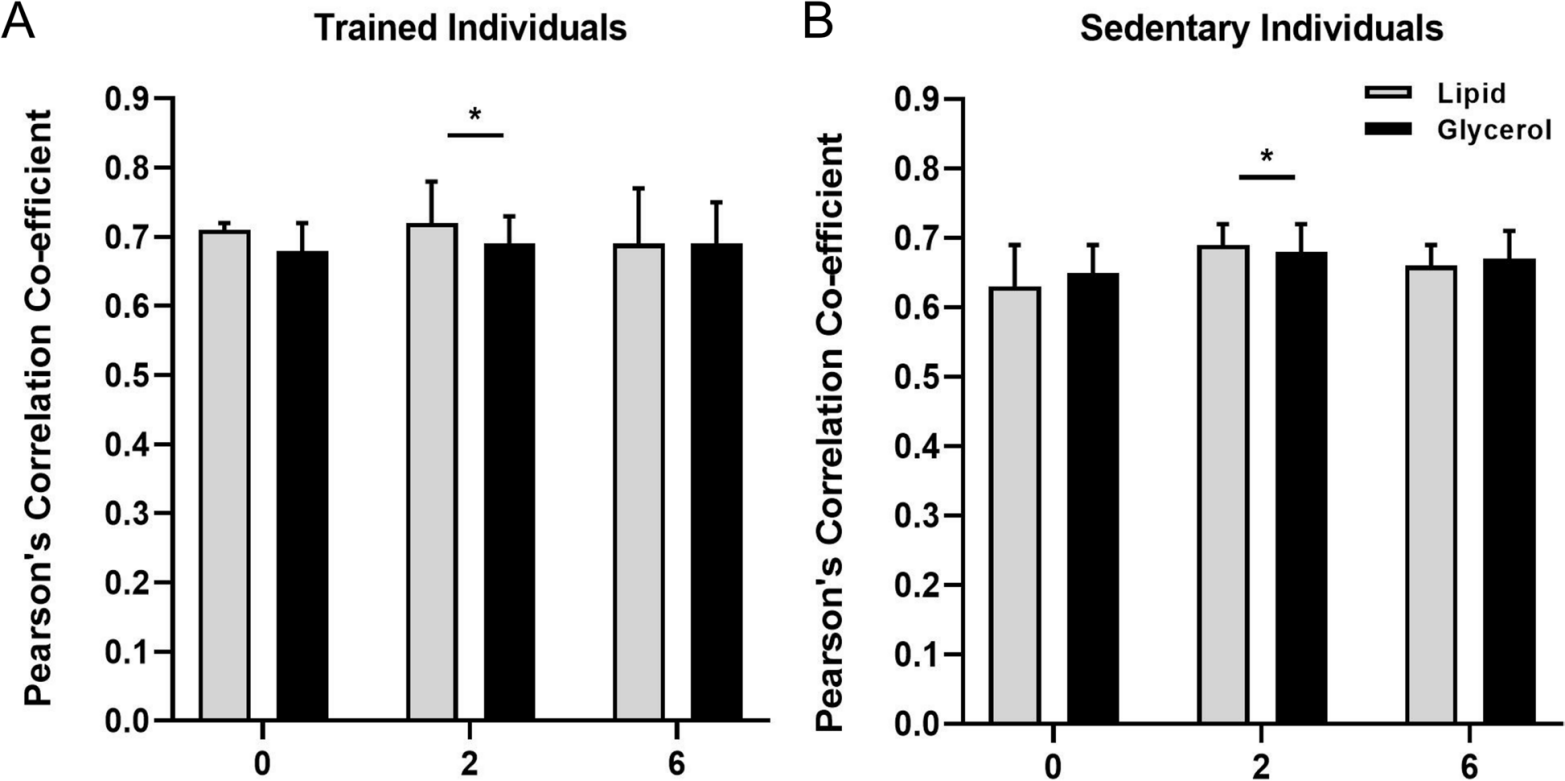
Colocalization of GLUT4 with PM marker dystrophin, measured using the Pearson’s correlation coefficient using linear mixed model. * Main time effect *P* = 0.039, with greater PCC at 2 h vs 0 h of infusion. There was no difference between 2 and 6 h (*P* = 0.557) or 0 and 6 h (*P* = 0.522)

Using immunofluorescence microscopy, it was observed that at baseline total protein expression of GLUT4 was greater in type 2 fibres compared to type 1 fibres (*P* = 0.011) but was not different between trained and sedentary individuals (*P* = 0.477; Fig. 3). Pearson’s correlation coefficient was used to determine the relative localisation of GLUT4 with the dystrophin stain (i.e., the plasma membrane), and was greater in trained individuals compared to the sedentary group at baseline (main training status effect; *P* = 0.020). Importantly, co-localisation was not different between infusion groups (*P* = 0.909; Fig. 4). GLUT4 fluorescence intensity was used as a marker of GLUT4 protein expression in the plasma membrane and the 5 intracellular layers, and at baseline GLUT4 fluorescence intensity was greatest in the plasma membrane compared to all intracellular layers (main effect of layer; *P* < 0.001).

### Visualisation of large and small GLUT4 spots at baseline

Using immunofluorescence staining we identified both larger spots of GLUT4 as well as small spots which are dispersed throughout the cell (Fig. 2d and e). To distinguish between large and small spots, threshold limits were used on all detected spots such that they were separated into large (defined as having a diameter of > 1 μm) and small spots (defined as having a diameter < 1 μm). These limits have been used previously in a number of studies by both ourselves and other research groups [11, 15, 25]. At baseline, the total number of GLUT4 spots in the plasma membrane was greater compared to all other intracellular layers (*P* < 0.001; Fig. 7), and this was also true for both large GLUT4 clusters (*P* < 0.001) and small GLUT4 spots (*P* < 0.001). Overall, the total number of GLUT4 spots was significantly greater at the plasma membrane in trained compared to sedentary (*P* < 0.001). Interestingly though, sedentary individuals had a greater number of large GLUT4 clusters at the plasma membrane compared to trained individuals (layer × training status; *P* = 0.001; Fig. 7), whereas trained individuals had a greater number of small GLUT4 spots at the plasma membrane compared to sedentary individuals (training status × layer; *P* < 0.001). Small GLUT4 spots made up ~ 97% of all GLUT4 spots in trained individuals, and at the plasma membrane ~ 99% of spots in the plasma membrane were categorised as small spots in trained individuals, whereas only ~ 95% of GLUT4 spots in the plasma membrane were small spots in sedentary individuals. Across both groups, more large spots were observed at the plasma membrane in type 1 fibres compared to type 2 fibres (layer × fibre type; *P* < 0.001, Fig. 7), but there was no fibre type difference in the number of small GLUT4 spots at the plasma membrane. Infusion group had no effect on the number of large or small GLUT4 spots at baseline (small spots, *P* = 0.520; large spots, *P* = 0.590).

### GLUT4 content and localization following a lipid or glycerol infusion

**Fig. 5:**
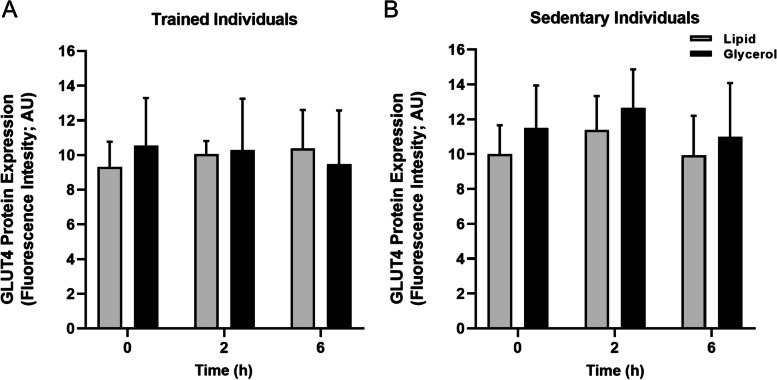
Total GLUT4 fluorescence intensity following the lipid (grey) and glycerol infusion (black) in trained and sedentary individuals

We next investigated whether there were time-dependent changes in GLUT4 localization in response to either a lipid or glycerol infusion. First though, we checked for any time-dependent changes in protein expression (using immunofluorescence microscopy) and found that GLUT4 protein expression (measured as GLUT4 fluorescence intensity) did not change over time (*P* = 0.062; Fig. 5) and was not different between infusion groups (*P* = 0.389) or trained and sedentary individuals (P = 0.380) at any time point.

Using Pearson’s correlation coefficient, we observed a small but significant increase in colocalization of GLUT4 with the plasma membrane from 0 to 2h of infusion (+ 2% in trained following both lipid and glycerol infusions, + 9% in sedentary after lipid infusion and + 4% after glycerol infusion, main time effect; *P* = 0.039; Fig. 4) and this remained elevated from 2 to 6h (*P* = 0.557). Notably though, there was no significant difference in GLUT4 colocalization with the plasma membrane between 0 and 6h of infusion (*P* = 0.522). GLUT4 fluorescence intensity within the plasma membrane was greater compared to all intracellular layers (main layer effect; *p* < 0.001) at both 2h and 6h of infusion, but GLUT4 fluorescence intensity did not change significantly over time in the plasma membrane (*P* = 0.071). Importantly, there was no difference between the infusion groups or trained and sedentary individuals when examining GLUT4 localization to the plasma membrane, or GLUT4 fluorescence intensity in the plasma membrane or intracellular layers.

### Changes in GLUT4 spots following a lipid or glycerol infusion

**Fig. 6:**
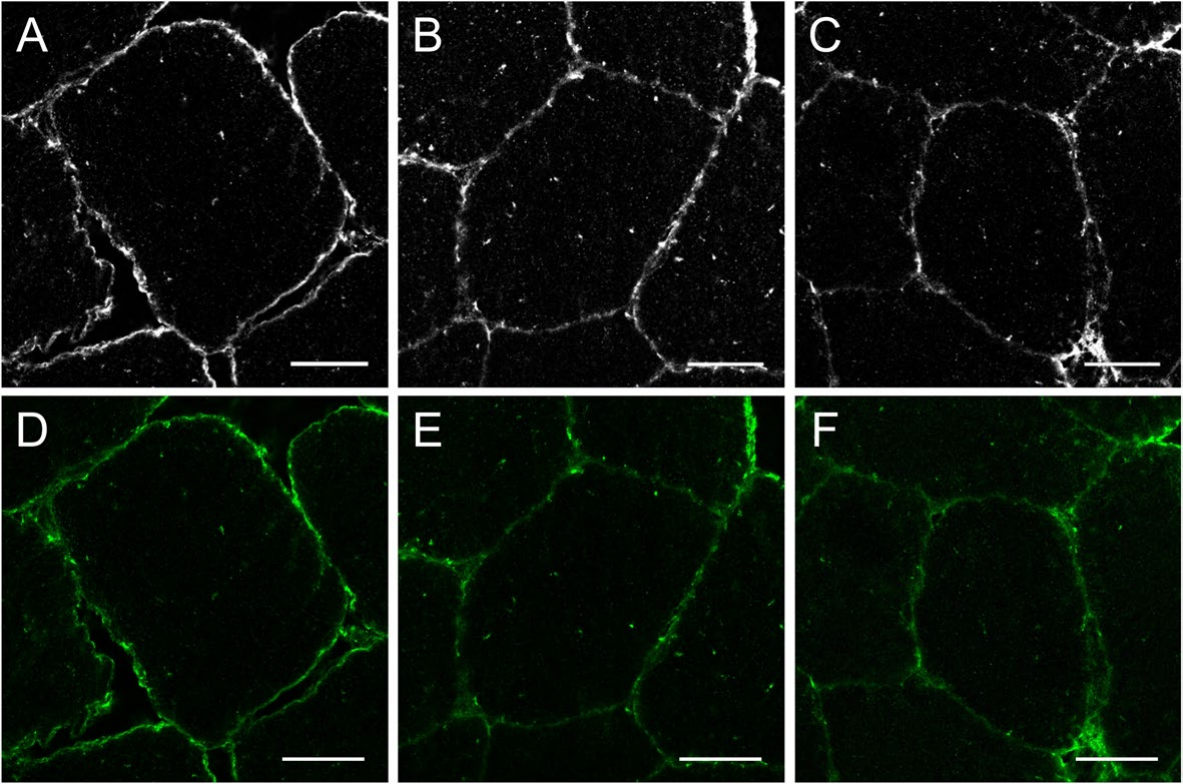
Representative immunofluorescence microscopy images demonstrating the GLUT4 stain (**A**, **B** and **C** are greyscale versions of **D**, **E** and **F**) from baseline (**A** and **D**), 2 h (**B** and **E**) and 6 h (**C** and **F**) following an intralipid infusion. Scale bar represents 25 μm

**Fig. 7:**
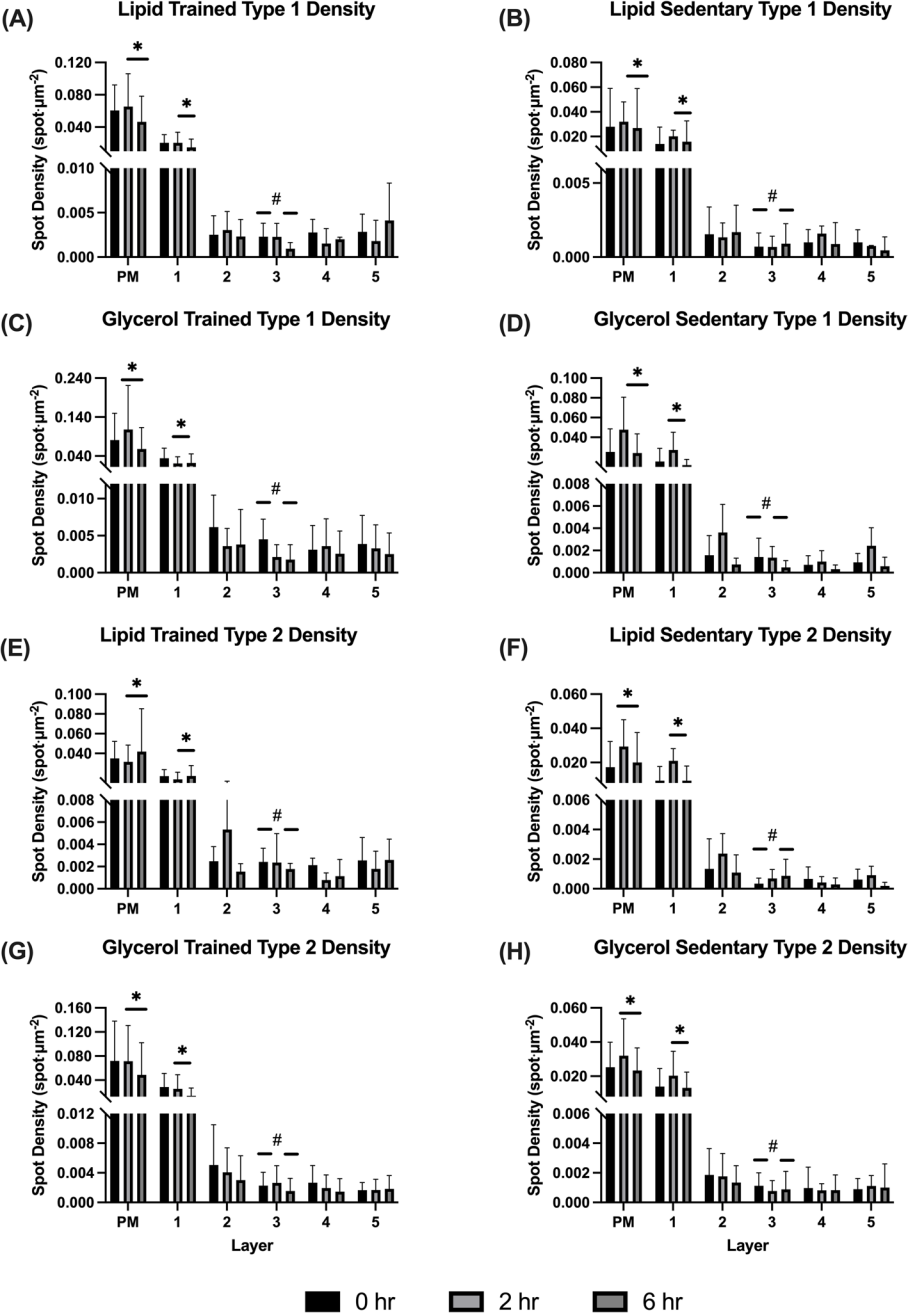
Average number of small GLUT4 spots in the PM and 5 intracellular layers at baseline and after 2 & 6h of a lipid (**A**, **B**, **E**, **F**) or glycerol (**C**, **D**, **G**, **H**) infusion in type 1 (**A**-**D**) and type 2 fibres (**E**–**H**) and in trained (right column) and sedentary individuals (left column). M = Linear mixed model * represents a decrease in the number of GLUT4 spots from 2 to 6h (PM; *P* = 0.047, layer 1; *P* = 0.045). ^#^ Represents greater number of GLUT4 spots at 0h compared to 6h in layer 3 (*P* = 0.038)

Changes in GLUT4 spots (see Fig. 2d and 2e for spot identification) were analysed within the plasma membrane and all 5 intracellular layers at all three timepoints. When considering the number of small GLUT4 spots, we observed a similar pattern as described above in the plasma membrane and layer 1 (Fig. 6), where there was no change in the number of small GLUT4 spots from 0 to 2h (PM; *P* = 0.486, layer 1; *P* = 0.669; Fig. 7), but a reduction in small GLUT4 spots from 2 to 6h (PM; *P* = 0.047: layer 1; *P* = 0.045). There was no difference between 0 and 6h in these two layers for number of small spots (PM; *P* = 0.820, layer 1; *P* = 0.550). Again, the same pattern was seen in layer 3 for the number of small GLUT4 spots with a decrease from 0 to 6h (*P* = 0.038), but no difference between 0 to 2h (*P* = 1.000) or 2 to 6h (*P* = 0.066). In layers 2, 4 and 5, there was no effects of time on the average number of small GLUT4 spots (layer 2; P = 0.097, layer 4; *P* = 0.093, layer 5; *P* = 0.705).

In contrast, the number of large GLUT4 spots did not change over time in the plasma membrane (*P* = 0.809) or layer 1 (*P* = 1.000). However, layers 4 and 5 saw changes in the number of large GLUT4 spots. In layer 4 there was a significant decrease in the number of large GLUT4 spots from 2 to 6h of infusion (*P* = 0.030), with no change between 0 to 2h (*P* = 1.000) or 0 to 6h (*P* = 0.287). In layer 5, there was a reduction in the number of large GLUT4 spots from 0 to 2h of infusion (*P* = 0.004), with no changes between 2 and 6h (*P* = 0.660) or 0 and 6h (*P* = 0.126).

## Discussion

The aim of present study was to examine the effects of acute FFA elevation (via infusion of intralipid®) alongside a hyperinsulinaemic euglycemic clamp on fibre type specific subcellular GLUT4 distribution in human skeletal muscle of sedentary and trained individuals. The novel findings of the study are that 1) GLUT4 localisation with the plasma membrane is unaffected by an increase in plasma FFA availability induced by lipid infusion, and 2) changes in GLUT4 spot number occurs independent of a change in GLUT4 fluorescence intensity.

### Training status dictates the number of small and large GLUT4 clusters

At baseline, we observed greater GLUT4 protein expression in type II fibres compared to type I fibres (~ 7% in trained individuals, ~ 12% in sedentary). Using the same technique, our laboratory has previously reported no difference in GLUT4 expression between fibre types, although this was only in well-trained individuals [25]. Notably, in the current study we also observed that total GLUT4 protein expression was comparable between trained and sedentary individuals. A well-known adaptation to exercise training is an increase in total skeletal muscle GLUT4 protein content [25, 27–37], and therefore we expected GLUT4 protein expression to be greater in trained compared to the sedentary individuals in the present study. It may be that the training status of our trained and sedentary participants was not sufficiently distinct to result in a significant difference in GLUT4 protein expression between groups (VO_2max_ ~ 49 ml/kg/min in the trained individuals vs. ~ 39 ml/kg/min in the sedentary individuals).

Concurrent with previous data from Bradley et al*.,* (2014 & 2015), GLUT4 in muscle was primarily located at the plasma membrane compared to all intracellular layers (Fig. 6). GLUT4 exists in clusters, which have previously been characterized as being small (< 1 μm) or large (> 1 μm) by ourselves [15, 25] and others [9–12]. Interestingly, trained individuals had a greater number of small GLUT4 spots in the plasma membrane, whereas sedentary individuals had more large GLUT4 spots. Small GLUT4 spots made up ~ 97% of all GLUT4 spots in trained individuals, and this explains why trained individuals had a greater total number of GLUT4 spots compared to sedentary individuals. Small GLUT4 spots have been identified as endosomal stores or glucose storage vesicles and are more mobile than the large GLUT4 spots [11] that are present in the membrane of the Trans-Golgi network [9, 10]. Thus, a greater number of small GLUT4 spots means that trained individuals have a larger pool of more mobile GLUT4. This difference in the number of small GLUT4 spots likely has implications for the translocation and cycling of GLUT4 at the plasma membrane, and subsequently support the greater rates of glucose uptake into muscle that are characteristic of trained individuals.

### GLUT4 localisation following lipid or glycerol infusion

When a hyperinsulinaemic euglycemic clamp is combined with infusion of lipid-heparin, both classical and contemporary research has demonstrated that insulin-stimulated glucose uptake is normal following 2 h of lipid infusion compared to a non-lipid control [38–40]. By 6 h of a lipid-heparin infusion though, insulin-stimulated glucose uptake is reduced compared to the control condition [38, 39]. On this basis, we hypothesized that increased GLUT4 co-localisation with the plasma membrane would be apparent at 2 h of infusion of either lipid or glycerol, but at 6 h of infusion there would be a divergence where GLUT4 co-localisation at the plasma membrane would be maintained following the glycerol infusion but reduced following the lipid infusion. Consistent with the first part of this hypothesis, we observed a significant increase in Pearson’s Correlation Coefficient following 2 h of both the lipid and glycerol infusion, which we interpret as increased insulin-stimulated GLUT4 localisation to the plasma membrane.

The increase in GLUT4 localisation at the plasma membrane was maintained at 6 h of glycerol infusion, as evidenced by the similar Pearson’s correlation coefficient values. Interestingly though, and in contrast to the second part of our hypothesis, we observed no change in GLUT4 co-localization with the plasma membrane from 2 to 6 h of the lipid infusion. We expected a decrease in GLUT4 at the plasma membrane at 6 h of lipid infusion to explain the previously reported reduction in glucose disposal [21]. Since there was no difference in GLUT4 co-localisation between 0 and 6 h of infusion, we could speculate that there may have been a small but non-significant reduction in GLUT4 localisation with the plasma membrane from 2 to 6 h. Nevertheless, we would only expect any reduction to occur in the lipid infusion group, but we observed the same effect in the glycerol infusion group. Taken together, our results suggest that GLUT4 localisation with the plasma membrane was unaffected by an increase in plasma FFA availability induced by lipid infusion. Insulin-stimulated glucose uptake is dependent, in part, on the insulin signalling cascade where activation of IRS-1 and Akt are critical. However, lipid infusion does not appear to decrease IRS-1 and Akt phosphorylation compared to control conditions over the same time-course [21, 22]. Therefore, it is perhaps not surprising that GLUT4 localisation to the plasma membrane was unchanged from 2 to 6 h of a lipid infusion. This suggests that the mechanisms by which increased FFA availability leads to a reduction in glucose infusion rate are unrelated to activation of the insulin signalling cascade and GLUT4 localisation at the plasma membrane.

It should be acknowledged that GLUT4 localisation to the plasma membrane was possibly stimulated by other factors independent of insulin signalling. For example, in cultured myotubes IL-6 stimulates GLUT4 translocation to the sarcolemma via AMP-activated protein kinase activation [41], and circulating IL-6 concentrations are increased during a hyperinsulinaemic euglycaemic clamp [42]. Therefore, it is possible that GLUT4 localisation with the sarcolemma could have been induced via an insulin-independent mechanism, even in the face of elevated FFA availability. Unfortunately, we do not have plasma samples remaining to measure IL-6 concentrations. It is also important to note that there was no fibre type difference in the co-localisation of GLUT4 to the plasma membrane. This corresponds with previous literature concluding no significant relationship between GLUT4 protein content and fibre type [43]. Very recent data from Koh et al., (2021) further solidifies this with significant differences in glucose disposal during a hyperinsulinemic euglycemic clamp between lean and obese individuals, yet no relationship with fibre type composition [44]. Even the post-exercise insulin sensitizing effect is shown to be similar between fibre types [45].

### Changes in GLUT4 spots following lipid or glycerol infusion

Beyond the use of co-localisation analysis to examine changes in GLUT4 localisation with the plasma membrane, confocal immunofluorescence microscopy also enables the identification and quantitation of GLUT4 clusters (spots). Our laboratory has previously shown that these GLUT4 clusters are present at both the plasma membrane and intracellular locations [15, 25]. Here, we report that the number of GLUT4 spots at the plasma membrane or the 1 µm below the membrane (layer 1) did not change following 2 h of either the lipid or glycerol infusion compared to baseline. However, from 2 to 6 h of either infusion there was a significant decrease in the number of GLUT4 spots at the plasma membrane and layer 1. More specifically, the reduction in total GLUT4 spots in the plasma membrane and layer 1 from 2 to 6 h of infusion could be entirely accounted for by a decrease in small GLUT4 spots (< 1 μm diameter). The decrease in GLUT4 spots occurred independent of a change in GLUT4 fluorescence intensity (and therefore total GLUT4 protein) within the plasma membrane, and therefore we speculate that in response to prolonged insulin infusion GLUT4 disperses from the storage vesicles (spots) in the plasma membrane. This proposed mechanism has similarities to that previously observed within adipocytes, whereby under basal conditions GLUT4 is retained in clusters at, or in close proximity to the plasma membrane, but upon insulin stimulation GLUT4 is then dispersed into the plasma membrane [46]. Although in our study this dispersal of GLUT4 is occurring in response to continuous insulin stimulation, it has previously been shown this is not sufficient to maintain glucose uptake into skeletal muscle when lipid is infused [21]. It appears that the suppression of glucose uptake in response to a lipid infusion is therefore not due to GLUT4 availability at the plasma membrane.

It is noteworthy that we also report a reduction in the number of large GLUT4 spots in layers 4 and 5. One possibility is that these reductions in large GLUT4 clusters at the more intracellular layers could suggest that this pool of GLUT4 is moving towards the membrane or layers 1, 2 and 3. However, no change in the number of large GLUT4 spots in these layers close to, or at the plasma membrane, were observed. It may be that these large GLUT4 clusters are dispersing and becoming small GLUT4 spots, in the same or different layers and may explain why we do not see a difference in the number of small GLUT4 spots in layers 3 and 4. Alternatively, these large spots may be reducing in size and at the same time, what were identified as small spots may be reducing in size beyond the limits of our detection, explaining a reduction in the number of large spots with no change in the number of small spots.

### Potential alternative mechanism for reduced glucose uptake

The long chain acyl-CoA’s palmitoyl-CoA, oleoyl-CoA and linoleoyl-CoA have been shown to inhibit hexokinase activity in rat and human skeletal muscle [47]. The impact of this inhibition is potentially a reduction in G6P concentrations, a reduction in glycogen synthesis and glycolysis, and ultimately a lower flux through hexokinase at lower G6P concentrations previously observed in insulin-resistant skeletal muscle (Rotheman et al*.,* 1992; Rothman et al*.,* 1995; Roden et al*.,* 1996; Jucker et al*.,* 1997; Petersen et al., 1998; Roden et al*.,* 1999). The well known Randle cycle demonstrates how increased lipid oxidation can result in reduced glucose uptake through pyruvate dehydrogenase and phosphofructokinase inhibition increasing G6P concentrations and inhibiting hexokinase [48]. LCA-CoA inhibition of hexokinase likely occurs simultaneously to the glucose-fatty acid cycle interaction and reduces insulin-stimulated glucose uptake producing an insulin-resistant state [47], often seen following lipid infusion in previous research [21, 22]. Therefore, continuous insulin stimulation likely supports the increased GLUT4 co-localisation and subsequent dispersal of GLUT4 at the plasma membrane in both the lipid and glycerol infusion conditions. However, when lipid is infused, the elevated FA availability will suppress glucose uptake via the inhibition of hexokinase explaining the reduction in glucose infusion rate previously reported [47]. Without the increase in FA, the glycerol infusion sees the same level of insulin stimulation, without the inhibition of hexokinase, and so GLUT4 can support glucose uptake.

### Strengths and limitations

In the present study, participants within each training status group were matched for BMI for the infusion group allocations, and importantly, trained and sedentary individuals were discrepant in VO_2max_. All fibres imaged were selected at random and not by inspection of GLUT4 stain. The membrane stain is used to isolate the cell, which may risk missing some of the GLUT4 that is at the plasma membrane from analysis. Previously, GLUT4 protein content had been measured in whole muscle samples and does not change over time in response to lipid infusion in men or women [49]. Whilst image analysis of individual muscle fibres is useful for changes in GLUT4 within the cell, using the border to isolate the cell from the rest of the image may lead to missing information at the plasma membrane.

The analysis method utilised in the present study limits our ability to make a comprehensive exploration of the different subcellular GLUT4 pools as seen in recent research [50]. Knudsen et al. utilised a technique they termed Sample Thinning Enhanced Resolution Microscopy (STERM), to visualise GLUT4 distribution throughout the endomembrane. This method required cutting ultra-thin biopsy sections to allow antibody penetration in the absence of detergent. The STERM method paired with the standard confocal microscopy workflow markedly improved the ability to resolve GLUT4 present in small vesicles from larger membrane structures. By then visualizing GLUT4 in STERM-prepared human muscle samples using transmission electron microscopy, Knudsen also confirmed GLUT4 localisation to cytosolic perinuclear, intramyofibrillar and subsarcolemmal areas, tubulovesicular structures, multivesicular endosomes and, most critically, small vesicles sized ~ 70—150 nm, some of which would presumably be detergent-sensitive [50].

Interestingly, it is evident that the degree of localisation of GLUT4 with the plasma membrane under resting conditions appears to be smaller than when exercise precedes insulin-stimulation [15, 50]. Exercise appears to be a stronger stimulus to induce GLUT4 translocation in skeletal muscle than insulin (~ 19% vs 9% increase in localisation), also suggested by Bradley et al. [15].

## Conclusions

The present data suggest that GLUT4 co-localisation is not significantly decreased following a lipid infusion when compared to a glycerol control. Decreases in GLUT4 spot number irrespective of GLUT4 intensity demonstrate that dispersal from clusters at the plasma membrane may facilitate glucose homeostasis.

## Data Availability

Corresponding authors may provide data upon request.
